# Wrangling Galaxy’s reference data

**DOI:** 10.1093/bioinformatics/btu119

**Published:** 2014-02-28

**Authors:** Daniel Blankenberg, James E. Johnson, The Galaxy Team, James Taylor, Anton Nekrutenko

**Affiliations:** ^1^Department of Biochemistry and Molecular Biology, Penn State University, University Park, PA 16802, USA, ^2^http://www.galaxyproject.org, ^3^Minnesota Supercomputing Institute, University of Minnesota, Minneapolis, MN 55455, USA, ^4^Department of Biology and ^5^Department of Mathematics and Computer Science, Emory University, Atlanta, GA 30322, USA

## Abstract

**Summary:** The Galaxy platform has developed into a fully featured collaborative workbench, with goals of inherently capturing provenance to enable reproducible data analysis, and of making it straightforward to run one’s own server. However, many Galaxy platform tools rely on the presence of reference data, such as alignment indexes, to function efficiently. Until now, the building of this cache of data for Galaxy has been an error-prone manual process lacking reproducibility and provenance.

The Galaxy Data Manager framework is an enhancement that changes the management of Galaxy’s built-in data cache from a manual procedure to an automated graphical user interface (GUI) driven process, which contains the same openness, reproducibility and provenance that is afforded to Galaxy’s analysis tools. Data Manager tools allow the Galaxy administrator to download, create and install additional datasets for any type of reference data in real time.

**Availability and implementation:** The Galaxy Data Manager framework is implemented in Python and has been integrated as part of the core Galaxy platform. Individual Data Manager tools can be defined locally or installed from a ToolShed, allowing the Galaxy community to define additional Data Manager tools as needed, with full versioning and dependency support.

**Contact:** dan@bx.psu.edu. or anton@bx.psu.edu

**Supplementary information:** Supplementary data is available at *Bioinformatics* online.

## 1 INTRODUCTION

Galaxy (Blankenberg *et al.*, 2010; Giardine *et al.*, 2005; Goecks *et al.*, 2010) is a web-based platform for performing large-scale data analysis. It is a completely open-source project that supports accessible, reproducible and transparent computational research and is available through the use of free public servers, private local installations and by launching instances in the Cloud. At the heart of Galaxy is its ability to integrate disparate data sources and analysis tools into a unified interface. Galaxy comes prepackaged with a default set of analysis tools, but additional tools can be defined locally or installed from a community-curated resource known as the Galaxy ToolShed (https://usegalaxy.org/toolshed). When a tool is executed within Galaxy, all of the user’s selections and parameters are recorded, providing provenance and enabling reproducible data analysis. When executing a tool installed from the Galaxy ToolShed, not only are input parameters recorded, but specific tool and dependency versions are also controlled; this enables reproducibility across time and between different Galaxy instances.

One weakness in this reproducibility is the reliance of many tools on built-in reference data, such as reference genome sequences or short-read mapper indexes (see Supplementary Figure S1). Until now, Galaxy administrators have been responsible for downloading, building and installing these important reference data. For example, to make the UCSC hg19 build of the human reference genome available to the Burrows-Wheeler Aligner (BWA) short-read mapper (Li and Durbin, 2009), a Galaxy administrator would need to (i) download the reference genome FASTA file, (ii) make it available as a reference genome via the ‘all_fasta’ table (optional), (iii) build BWA alignment indexes via proper command-line calls, (iv) register the location and availability of the indexes within the ‘bwa_indexes’ data table (by adding an additional entry to the tool-data/bwa_index.loc file on disk) and (v) finally, restart the Galaxy server. Although not technically challenging, each one of the previously mentioned manual steps is prone to error and lacks any provenance; any incorrectness or incompleteness of built-in data will have a severe impact on the correctness of a subsequent analysis. Worse yet, it may not even be apparent that something has been configured incorrectly, creating a situation where invalid results are trusted.

Data Manager tools remove the technical burdens of ensuring the reproducibility and provenance of built-in reference data from the hands of the Galaxy administrator and make it an automated point-and-click process. A new menu option, ‘Manage local data’, has been added to the Galaxy administrator interface. Accessing this option enables an administrator to run Data Manager tools, inspect the results of individual Data Manager executions and view the current state of Galaxy’s built-in data registries. Running a Data Manager uses the same familiar interface as a standard Galaxy tool, allowing an administrator to configure the Data Manager with desired options (e.g. dbkey, source reference FASTA file, indexing algorithm). On completion of a Data Manager tool run, the Data Manager framework parses the output for new data table entries and values. These values are enabled in real time and persisted to disk. Restarting the Galaxy server is not required for enabling the new entries; however, the new entries will remain after a restart. Although the Data Manager framework negates the need for the manual curating of reference data, it is compatible with any previously existing policy or process in-use for a Galaxy installation.

## 2 METHODS

A Galaxy Data Manager is composed of two primary components: a Data Manager tool and a Data Manager configuration. Similar to standard Galaxy tools, the Data Manager tool component is responsible for defining the user configurable components as well as the command line and scripts used to generate the actual underlying data (e.g. download a FASTA genome, run the BWA binary to build index files). The Data Manager configuration component instructs the framework on how to process the output of the Data Manager tool into new entries into Galaxy’s built-in data registry. Although a Data Manager can add any number of new entries to any number of data tables, the most common case is to add a single new entry to a single data table. Whereas the examples listed here involve reference genomes or reference genome indexes, it is worth noting that any type of preconfigured Galaxy data, such as BLAST databases and protein or pathway domain databases, can be incorporated into a Data Manager.

### 2.1 Data Manager tools

Data Manager tools were implemented as an extension to standard Galaxy tools. A Galaxy tool can be loosely defined as being composed of two parts: (i) an XML-based tool description that defines the input parameters and settings, the manner in which to assemble a command line to be executed, the output files generated by the command line and on-screen help and (ii) the underlying command-line executable(s). The only difference between defining a standard Galaxy tool and a Data Manager tool is the inclusion of the type = ‘data_manager’ attribute to the <tool> element; this declaration has the affect of instructing Galaxy to provide a JavaScript Object Notation (JSON) encoded dictionary of parameter and server settings to be optionally used by the executable and to trigger the Data Manager framework to process the tool output into new data table entries. By design, Data Manager tools can only be executed by a Galaxy administrator. There is no requirement of the underlying executable to parse the input JSON, but using this JSON input can greatly simplify the command-line arguments that need to be defined and parsed by the underlying command-line executable. 

The only requirement of the underlying executable is that it provides as output a JSON-encoded description of the new entries to add to Galaxy’s built-in data registry. These values provided by the tool need not be the final values that will be stored, but may represent base values that will be modified by the Data Manager framework. At no point does the underlying executable need to interact directly with a data table or location file; entries are always created using this abstract communications layer. Often, the underlying executable is a wrapper script that handles parsing the input parameters, calling a set of binary executables and writing out the JSON-encoded new entry values. For example, the BWA indexer Data Manager calls a script that handles the tool input (e.g. determining the indexing algorithm to use: either declared explicitly by the user in the Galaxy interface or by guessing based on the size of the specified input genome), executes the ‘bwa index’ command and writes a JSON file containing the new data table entry values (a dictionary containing an ID, dbkey, display text and files path).

### 2.2 Data Manager configurations

Data Manager configurations are XML-based descriptions that define the actions of the Data Manager. Each configuration element specifies a single Data Manager, with name and id, the tool used by the Data Manager, the data tables that may be modified by this Data Manager, how to modify, if necessary, the raw entry values provided by the tool into the finalized values to be stored and how to handle the actual index files on disk (e.g. move a directory of files into the permanent built-in data store location). In the BWA example, the provided ID, dbkey and display text are used as-is, but the Data Manager framework modifies the file path value. Here, the path value provided by the executable is the FASTA file base name (e.g. genome_build.fa), which will be modified to match the final path on disk as configured by the administrator. These value modifications are handled using an extensible collection of value_translation tags.

## 3 CONCLUSIONS

The Data Manager framework represents a major improvement to Galaxy’s handling of built-in reference data. Previously, adding reference data to Galaxy was a manual process, relying entirely on an administrator to build reference data on the command line. It was solely the responsibility of the administrator to externally record the source of the data and the steps taken to prepare the data if provenance was desired. All correctness of the reference data or the manual addition of entries to location files required the careful attention of the administrator.

In addition to automating the administration of Galaxy’s built-in data cache, the Data Manager framework provides a pluggable approach for ensuring reproducibility and provenance tracking of reference data. By extending the capability of standard Galaxy tools, the Data Manager framework gains all the power that is available to the tool integration framework while providing a lower entry barrier to developers who are already familiar with defining Galaxy tools. The Data Manager framework is fully supported by the Galaxy ToolShed. New Data Manager tools can be defined by community members and installed into any Galaxy instance by the administrator. Particular attention has been paid to ensure that the configuration settings available will allow effective use of Data Manager tools across varying server environments and on the Cloud. Data Manager tools can be accessed using the GUI within Workflows or by using the application programming interface (API).

## Supplementary Material

Supplementary Data

Supplementary Data
