# Supplementary figures and images for: Wrangling Galaxy’s reference data
Source: Bioinformatics. 2014 Feb 28;30(13):1917–9. doi: 10.1093/bioinformatics/btu119 (PMC4071198; doi:10.1093/bioinformatics/btu119)

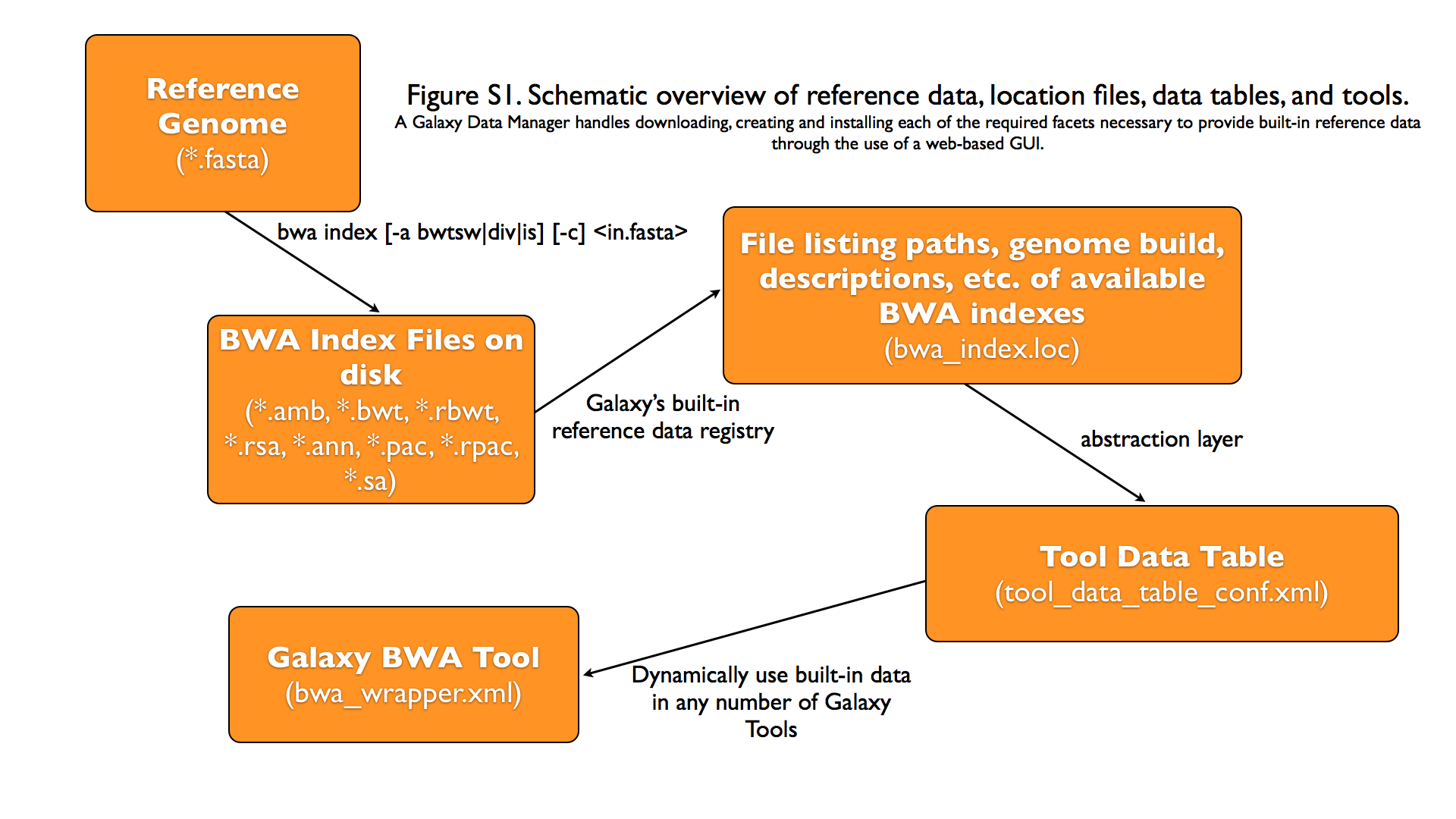

Supplement: Supplementary Data [file supp_btu119_figure_S1_schematic_overview.png]
