# Supplementary material for: Wrangling Galaxy’s reference data
Source: Bioinformatics. 2014 Feb 28;30(13):1917–9. doi: 10.1093/bioinformatics/btu119 (PMC4071198; doi:10.1093/bioinformatics/btu119)
Supplement: Supplementary Data [file supp_30_13_1917_v2_index.html]

Wrangling Galaxy’s reference data — Supplementary Data 

# Wrangling Galaxy’s reference data

## Supplementary Data

files

**Files in this Data Supplement:**

- Supplementary Data - png file
